# Spontaneous Subdural Hematomas as Initial Presentation of Acquired Coagulopathy: A Case Report

**DOI:** 10.7759/cureus.79246

**Published:** 2025-02-18

**Authors:** Natalia C Badillo-Velez, Yoan Rodriguez, Payam Nabizadeh-Eraghi, Jeffrey Bubis

**Affiliations:** 1 Internal Medicine, Hospital Corporation of America (HCA) Florida Orange Park Hospital, Orange Park, USA; 2 Internal Medicine, Orange Park Medical Center, Orange Park, USA; 3 Pulmonary and Critical Care Medicine, Hospital Corporation of America (HCA) Florida Orange Park Hospital, Orange Park, USA; 4 Hematology and Oncology, Florida Cancer Specialists and Research Institute, Jacksonville, USA

**Keywords:** acquired hemophilia a, case report, rheumatoid arthritis, spontaneous subdural hematoma, subdural hematoma

## Abstract

The evaluation of coagulopathies can be challenging due to the extensive variety of etiologies. This can be particularly complicated in patients with concomitant autoimmune disorders. In this case, a 64-year-old woman presented with intractable headaches and neck pain for two weeks. Her coagulation tests revealed an isolated prolongation of activated partial thromboplastin time (aPTT). This case highlights the diagnostic approach and summarizes management considerations in patients with similar presentations.

## Introduction

Acquired hemophilia A (AHA) is a rare condition triggered by an autoantibody targeting factor VIII (FVIII). In congenital hemophilia A, FVIII-neutralizing inhibitors are alloantibodies that arise after FVIII replacement therapy, whereas in AHA, these inhibitors are autoantibodies. Evaluations of various populations have estimated an annual incidence of acquired hemophilia A to be roughly one to two cases per million [1,2]. The predominant observation in about 80 percent of cases is subcutaneous bleeding [3]. Symptomatic individuals typically exhibit extensive ecchymoses, large hematomas, and severe mucosal bleeding, which may lead to medical emergencies. In a comprehensive survey involving 215 patients, 87 percent encountered major bleeding episodes, with complications linked to the inhibitor resulting in a 22 percent mortality rate [4]. In previous studies, the overall mortality rates have ranged from 31% to 33% [5,6].

Even though AHA affects individuals of all ages and genders, pregnant patients and older adults (>60 years old) experience a higher burden [7,8]. The European Acquired Hemophilia Registry (EACH2) indicated that AHA was idiopathic in 51.9% of cases, while malignancy or autoimmune diseases were associated with 11.8% and 11.6% of cases, respectively [9]. Intracranial bleeds have been scarcely reported for this condition in the literature [10-12]. Here, we present a case of a patient with acquired hemophilia A who developed spontaneous acute subdural hematomas in the setting of long-standing rheumatoid arthritis. The patient was effectively treated with steroids, recombinant factor VIIa, and rituximab.

## Case presentation

A 64-year-old Hispanic female with a history of longstanding rheumatoid arthritis was evaluated in the emergency department for headaches not relieved by over-the-counter analgesics. The headaches progressed with increasing severity over two weeks. She explained that symptoms began slowly and progressed to severe, hour-long episodes of sharp posterior headache radiating to her neck. The patient noted new-onset ecchymoses in her feet and left upper extremity, lasting for one week. These changes were previously attributed to her rheumatoid arthritis medication given normal basic laboratory assessment. The patient denied anticoagulation therapy use or a family history of hematologic disorders. She had no other coagulopathy symptoms, including GI bleeds, epistaxis, or hemoptysis. Neurologic examination was unremarkable with no associated migrainous features or postural component. The patient was previously taking prednisone 10 mg daily, diclofenac 75 mg (twice a day) BID, and methotrexate 25 mg weekly (she self-discontinued the methotrexate one week prior to admission) for rheumatoid arthritis. Rituximab was once prescribed given the severity of the symptoms; nonetheless, the patient self-discontinued it more than a year before due to concern for potential side effects.

**Figure 1:**
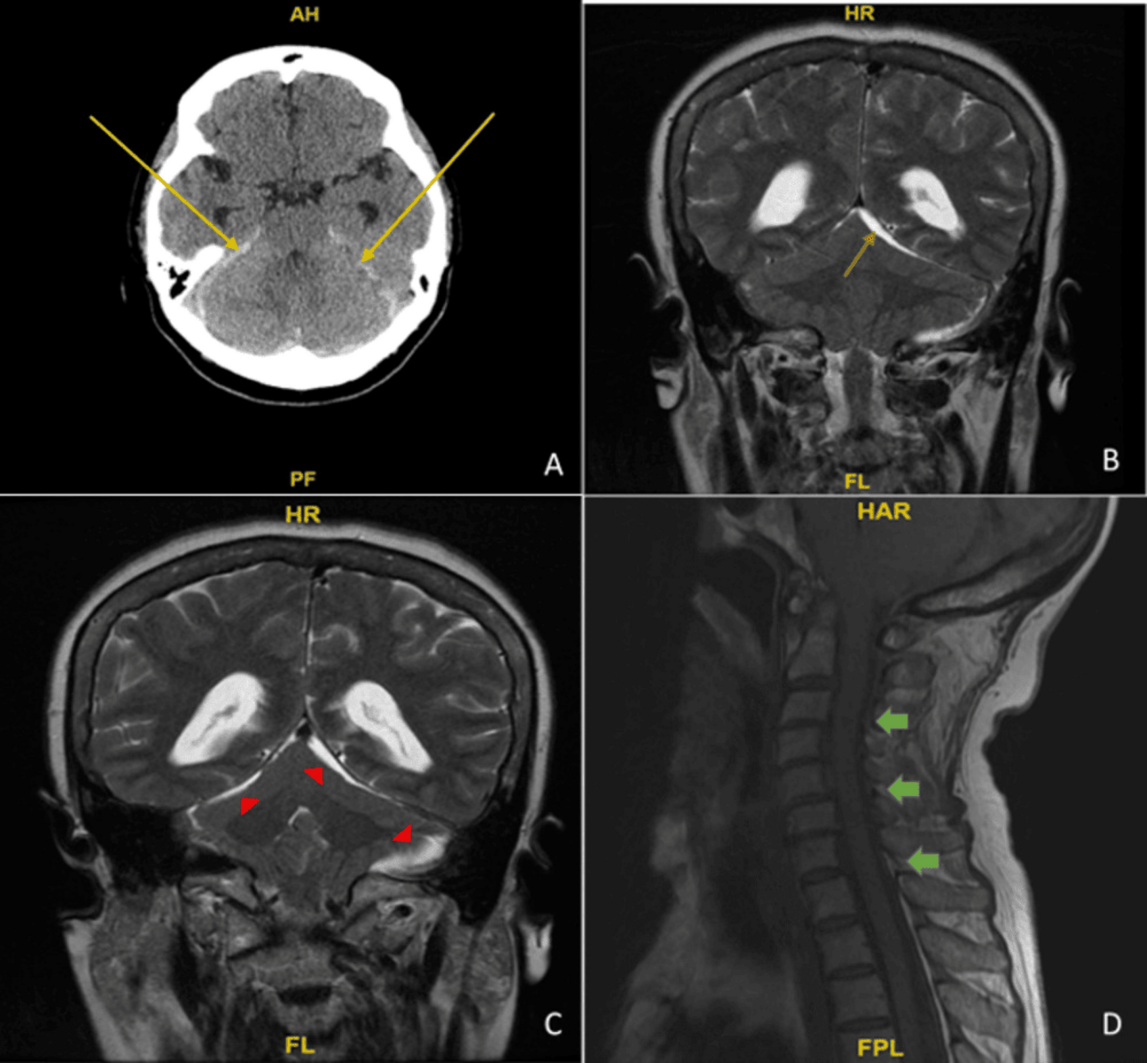
Computed tomography (CT), brain magnetic resonance image (MRI), and cervical spine MRI on admission A: brain CT shows acute subdural blood in the posterior fossa and bilateral tentorium, measuring 5 mm, with crowding of the foramen magnum and suprasellar cisterns as indicated by the yellow arrows; B: T2 coronal brain MRI shows hemorrhagic debris in the tentorium and peripheral aspect of the posterior fossa with inferior displacement or ectopia of the cerebral tonsils highlighted by the orange arrow; C: in the corresponding areas as shown by red arrowheads; D: cervical spine MRI without contrast sagittal T1 demonstrates no significant spinal hemorrhage as demonstrated by green arrows.

Given persistent symptoms, a brain CT scan was performed, revealing acute subdural blood in the posterior fossa and bilateral tentorium, measuring 5 mm, with crowding of the foramen magnum and effacement of the suprasellar cisterns (Figure 1A). Brain MRI was significant for hemorrhagic debris (Figure 1B, Figure 1C) and routine laboratory tests revealed an isolated elevation of partial thromboplastin time (PTT) at 85 and mild microcytic anemia. The chemistry panel, liver panel, and international normalized ratio (INR) were unremarkable. Further imaging comprised a CT angiogram, which showed no evidence of vasculitis or aneurysms, and a spinal MRI, revealing no significant spinal hemorrhage (Figure 1D). Furthermore, a vertebral/cerebral angiogram ruled out a dural arteriovenous fistula. The patient’s clinical picture was complicated by hematuria and oozing from the procedure site with a slow decrease in hemoglobin levels requiring transfusion of two units of leuko-reduced RBC and one unit of frozen plasma.

A clotting mixing study that failed to correct with a 1:1 ratio with plasma (indicating the presence of an inhibitor) and a nearly undetectable factor VIII level (consistent with AHA) was found upon further assessment. Factor IX was 107% (65 - 150%). Lupus anticoagulant testing was performed and showed anti-cardiolipin IgG of < 1.6 U/mL, normal levels of anti-cardiolipin IgM < 1.6 U/mL, and normal levels of anti-cardiolipin IgA < 2.0 U/mL. The rheumatoid factor screen was significantly elevated at 315.4 lnU/mL (0.0 - 12.0 lnU/mL). Laboratory tests for infectious etiology were negative. The patient was started on recombinant activated factor VII (rFVIIa) and prednisone 60 mg PO daily. Headaches and nausea markedly improved with opioid therapy. In addition, the patient was treated with rituximab 600 mg/ml IV. Upon discharge, the patient’s symptoms improved significantly, and repeated imaging showed stable thin irregular subdural hematomas.

## Discussion

Bleeding has been described in AHA; however, presentation with atraumatic spontaneous subdural hematomas is rare. AHA should be suspected in patients who exhibit spontaneous bleeding without a history of trauma or previous bleeding episodes, normal platelet counts and PT values, and an isolated prolongation of the activated partial thromboplastin time (aPTT). Although lupus anticoagulants can cause aPTT prolongation, they usually do not cause the bleeding symptoms associated with AHA [13]. In this case, given the patient’s spontaneous tentorial subdural hematomas, an arteriogram was performed to rule out a dural arteriovenous fistula. This intervention led to oozing from the puncture site requiring transfusion of blood products. While evaluation of an atraumatic subdural hematoma may lead to a traditional angiogram, there is still uncertainty regarding the risk of bleeding after the procedure in patients with AHA and patients undergoing similar procedures such as lumbar punctures in other common coagulopathies [14]. Given the absence of findings suggestive of vascular malformation in MRA or CTA imaging, this case raises the question of whether angiograms should be avoided in similar future cases. If feasible, these procedures should be postponed until the inhibitor has been eradicated. Previously, international recommendations have suggested (Grade 1B) the prophylactic use of bypassing agents to cover minor or major invasive procedures [3]. 

Individuals with an acquired factor inhibitor should take plasma derivatives or coagulation factors, bypassing the inhibited factor for acute bleeding management. The first line of hemostatic therapy is inhibitor bypassing agents (rFVIIa and activated prothrombin complex concentrate (aPCC)), which are less effective than desmopressin or human factor VIII concentrate [8]. Furthermore, a 2020 International Guideline for treating AHA recommended recombinant porcine factor VIII as an alternative to bypassing agents. Conversely, emicizumab is a recombinant, humanized, bispecific monoclonal antibody with FVIII-mimetic activity that may be administered as prophylaxis to hemophilia A patients with or without factor VIII inhibitors to avoid or lessen the frequency of bleeding episodes [3]. Additionally, prompt initiation of immunosuppressive treatment should be considered to eradicate inhibitory autoantibodies to FVIII due to the risk of life-threatening consequences. In our patient’s case, we used steroids and rFVIIa in conjunction with rituximab with a successful short-term outcome. Rituximab, a monoclonal antibody targeting the B-cell CD20 antigen, has recently surfaced as a potentially effective novel treatment for inhibitor eradication in AHA. According to the recommendations, rituximab is a safe and efficient first- or second-line treatment when combined with cytotoxic or steroid therapy [15]. While guidelines support rituximab as a first- or second-line option in combination with steroids or cytotoxic therapy, further research is needed to determine if rituximab alone could be a safe and effective first-line treatment for patients with lower initial inhibitor titers. After the successful elimination of FVIII inhibitors, patients should be evaluated monthly for the initial six months, every two to three months for the following six months, and then every six months for one year for surveillance.

## Conclusions

Acquired hemophilia A is a rare hematologic disorder with a wide margin of presentations where the culprit is an autoantibody that inhibits factor VIII. It is associated with increased morbidity and mortality. Even though early diagnosis can be difficult in patients without a previous history of bleeding disorders, it is necessary to maintain a high index of suspicion, mainly in populations at risk, such as patients with mixed connective tissue disorders. In the case of spontaneous intracranial hemorrhage, isolation of aPTT prolongation should prompt immediate workup and immunosuppressive treatment. The management principles of AHA involve bleeding control, elimination of FVIII inhibitor, and treatment of comorbidities, particularly, those that predispose to the development of FVIII inhibitor.
